# Predicting pathological complete response to neoadjuvant chemotherapy in breast cancer patients: use of MRI radiomics data from three regions with multiple machine learning algorithms

**DOI:** 10.1007/s00432-024-05680-y

**Published:** 2024-03-21

**Authors:** Guangying Zheng, Jiaxuan Peng, Zhenyu Shu, Hui Jin, Lu Han, Zhongyu Yuan, Xue Qin, Jie Hou, Xiaodong He, Xiangyang Gong

**Affiliations:** 1 Cancer Center, Department of Radiology, Zhejiang Provincial People’s Hospital (Affiliated People’s Hospital), Hangzhou Medical College, No. 158 Shangtang Road, Hangzhou, Zhejiang China; 2 Jinzhou Medical University, Jinzhou, Liaoning China

**Keywords:** Breast cancer, Radiomics, Neoadjuvant chemotherapy, Pathological complete response, Background parenchymal enhancement, Machine learning

## Abstract

**Objective:**

To construct a multi-region MRI radiomics model for predicting pathological complete response (pCR) in breast cancer (BCa) patients who received neoadjuvant chemotherapy (NACT) and provide a theoretical basis for the peritumoral microenvironment affecting the efficacy of NACT.

**Methods:**

A total of 133 BCa patients who received NACT, including 49 with confirmed pCR, were retrospectively analyzed. The radiomics features of the intratumoral region, peritumoral region, and background parenchymal enhancement (BPE) were extracted, and the most relevant features were obtained after dimensional reduction. Then, combining different areas, multivariate logistic regression analysis was used to select the optimal feature set, and six different machine learning models were used to predict pCR. The optimal model was selected, and its performance was evaluated using receiver operating characteristic (ROC) analysis. SHAP analysis was used to examine the relationship between the features of the model and pCR.

**Results:**

For signatures constructed using three individual regions, BPE provided the best predictions of pCR, and the diagnostic performance of the intratumoral and peritumoral regions improved after adding the BPE signature. The radiomics signature from the combination of all the three regions with the XGBoost machine learning algorithm provided the best predictions of pCR based on AUC (training set: 0.891, validation set: 0.861), sensitivity (training set: 0.882, validation set: 0.800), and specificity (training set: 0.847, validation set: 0.84). SHAP analysis demonstrated that LZ_log.sigma.2.0.mm.3D_glcm_ClusterShade_T12 made the greatest contribution to the predictions of this model.

**Conclusion:**

The addition of the BPE MRI signature improved the prediction of pCR in BCa patients who received NACT. These results suggest that the features of the peritumoral microenvironment are related to the efficacy of NACT.

**Supplementary Information:**

The online version contains supplementary material available at 10.1007/s00432-024-05680-y.

## Introduction

Breast cancer (BCa) is one of the most common malignant tumors in women (Houghton and Hankinson 2021; Giaquinto et al. 2022), and neoadjuvant chemotherapy (NACT) is a common treatment for patients with locally advanced BCa. The application of chemotherapy drugs before surgery reduces the size of the tumor, and this improves the feasibility of surgical resection and patient prognosis (Early Breast Cancer Trialists' Collaborative Group (EBCTCG) 2018). However, due to the heterogeneity and complexity of BCa, some patients do not respond well to NACT. Therefore, it is crucial to predict the effect of NACT in BCa patients so that the most appropriate treatment plan can be administered.

Patients with locally advanced BCa who achieve a pathological complete response (pCR) after NACT have a good prognosis, and research has therefore focused on predicting pCR after NACT in these patients (Montemurro et al. 2020). Radiomics, a method of extracting high-dimensional data from radiological medical images using different data representation algorithms (Mayerhoefer et al. 2020), is widely used in tumor diagnosis, treatment planning, and prognostic assessment. Thus, researchers have examined the use of radiomics from magnetic resonance imaging (MRI) data as a noninvasive method for predicting pCR after NACT in BCa patients. Early radiomics studies analyzed different types of primary tumors to predict pCR (Chen et al. 2020). The use of more sophisticated radiomics methods has shown that the radiomics features extracted from peritumoral tissue can also predict which patients will achieve a pCR before treatment (Braman et al. 2017). These results characterize the environment in which the tumor grows and can also help quantify disease evolution over the next few years and possibly predict tumor recurrence or progression (Gillies et al. 2002). However, it is difficult to identify and study the tissues in which tumors may grow in future.

Therefore, Gu et al. developed the concept of the peritumoral microenvironment (Gu et al. 2022) to characterize the mechanisms of tumorigenesis and progression. The peritumor microenvironment includes tissues that surround the tumor and are in proximity to the environment where the tumor will grow. Thus, tumor occurrence, recurrence, and metastasis all depend on the peritumoral microenvironment. Although peritumoral tissue includes regions with tumor cell infiltration and non-infiltrated normal tissue, the peritumoral microenvironment refers to non-tumor tissue that provides a suitable environment for tumor cell growth. Therefore, some studies of BCa have focused on background parenchymal enhancement (BPE) in breast magnetic resonance imaging (MRI) because it is more representative of the peritumor microenvironment (Wang et al. 2015). BPE is defined as a normal background enhancement of fibroglandular tissue after gadolinium injection (Rella et al. 2018; Telegrafo et al. 2016a) and is associated with physiological vascularization and perfusion of breast tissue (Wu et al. 2017; Brooks et al. 2018). There is evidence that a high BPE is associated with tumor malignancy, lymph node metastasis, and increased risk of recurrence (Telegrafo et al. 2016b). There is also evidence that a decrease in the BPE after chemotherapy is associated with a favorable response to NACT (Rella et al. 2020; Preibsch et al. 2016; La Forgia et al. 2021). Therefore, we hypothesized that the use of radiomic analysis of BPE tissues for characterization of the tumor and peritumoral microenvironment would provide improved predictions of pCR in women who receive NACT for locally advanced BCa.

This study examined the value of MRI radiomics features extracted from intratumoral, peritumoral, and BPE for prediction of pCR after NACT in patients with locally advanced BCa and also determined whether the combination of different tissue regions, especially the peritumoral microenvironment, can improve these predictions.

## Materials and methods

### Patient characteristics

**Fig. 1:**
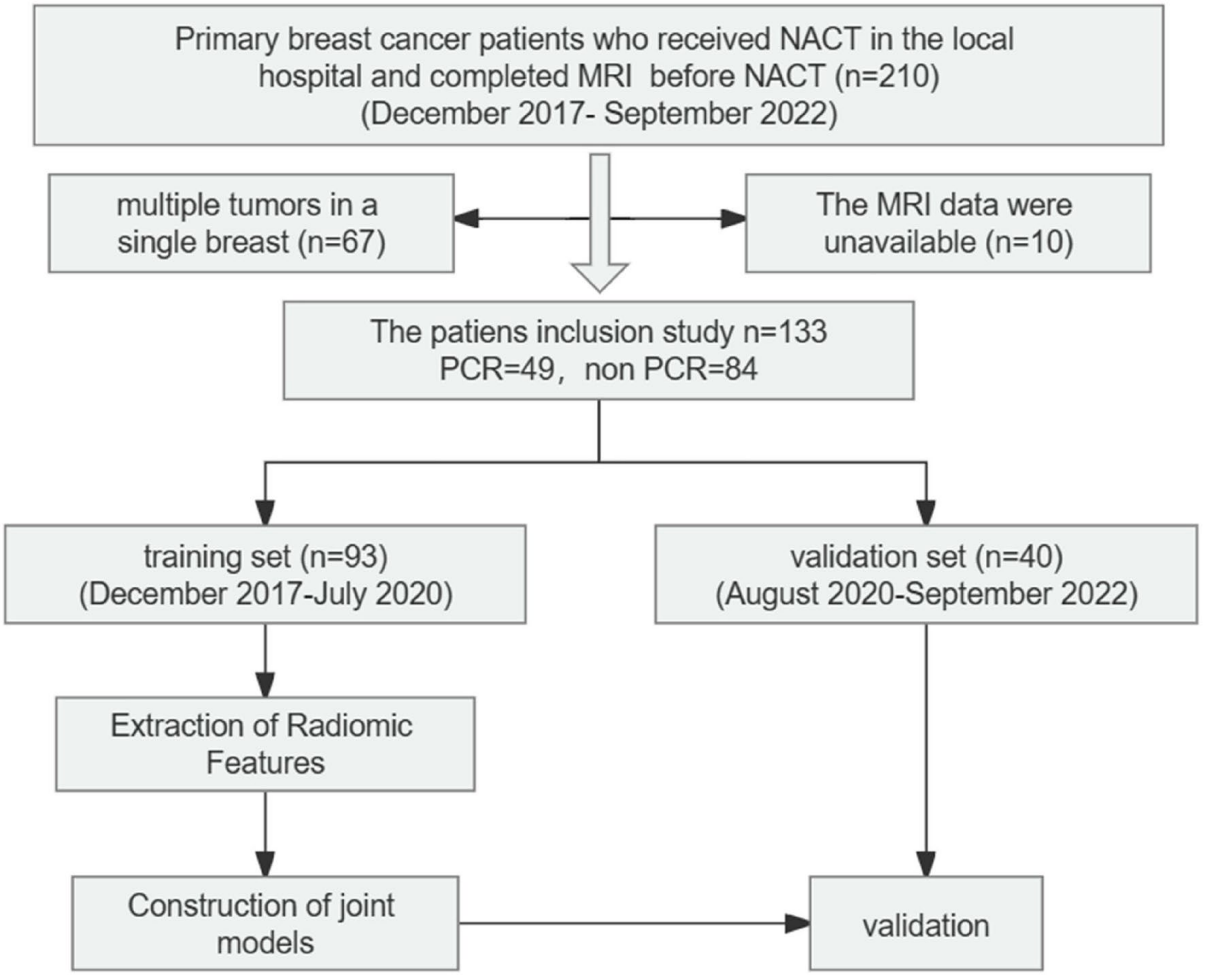
Disposition of retrospectively enrolled patients and overall study design

The study design was approved by the local institutional ethics committee, and the records of all patients were anonymized prior to data analysis. Due to the retrospective nature of the study, the institutional review board waived the need for written informed consent. Figure 1 shows the procedures used for patient selection and the overall experimental design. The inclusion criteria were: (a) presence of biopsy-proven primary invasive BCa without distant metastases; (b) completion of a standard NACT regimen, with no treatment prior to NACT; and (c) receipt of surgery after NACT followed by complete postoperative pathological evaluation. The exclusion criteria were: (a) no receipt of NACT, or receipt of a non-standard NACT regimen; (b) no surgery or surgery performed in another hospital; (c) unilateral BCa with multiple lesions; and (d) poor quality of MRI images.

All 210 patients with primary BCa who received NACT at our local hospital between December 2017 and September 2022 and underwent MRI before starting NACT were included. Ten patients with unavailable MRI results and 67 patients with multiple tumors in a single breast were excluded. Among the remaining 133 patients, the pathology results indicated that 49 had pCR status (Miller–Payne grade 5 and the absence of lymph node invasion in the ipsilateral sentinel node or lymph nodes removed during axillary dissection) and 84 had non-pCR status. These patients were randomly divided into a training group (*n* = 93) and a validation group (*n* = 40) in a 7:3 ratio. The Supplementary Materials provide details of the NACT protocol, Miller–Payne grading system, definition of pCR, immunohistochemical evaluations, and BCa subtypes.

### Image preprocessing and segmentation of regions of interest

All patients were scanned using a 3.0 T MRI scanner (Skyra; Siemens Healthineers) with a 16-channel body coil while in the prone position. T1-weighted images (T1WIs), third-phase enhanced T1-weighted (T1 + C) images, and dynamic contrast-enhanced subtraction images were recorded. A post-processing workstation was used to subtract the T1WI images from the third-phase enhanced T1-weighted (T1 + C) images to obtain subtraction images. The Supplementary Materials and Table S1 provide details of the imaging protocol and parameters. Before feature extraction, T1WI was used as a rigid registration template for all sequences. Image preprocessing with Matlab and SPM software, with registration of the T1WIs, T1 + C images, and silhouette images, was performed to ensure that the three sequences had the same resolution, spacing, and origin, by reducing the potential influence of scanning protocol parameters (https://www.fil.ion.ucl.ac.uk/spm/).

The standardized T1WIs were then imported into the open-source ITK-snap software (www.itksnap.org, version 3.8.0) to manually segment the entire tumor volume of interest (VOI) layer by layer. As described in previous studies, the peritumoral VOI was manually segmented around the tumor with a radius of 2.5 to 5 mm (Braman et al. 2017). Finally, the remaining normal breast tissue was segmented into regions with BPE. Two radiologists (one with 8 years and the other with 10 years of experience in BCa diagnosis) independently performed VOI delineation, and interobserver reproducibility was assessed. These two radiologists were blinded to clinical information and histopathological diagnosis. They independently segmented the images recorded prior to NACT in 20 randomly selected samples, and the features extracted from the above two VOIs from each of these 20 patients were compared using the intra-class correlation coefficient (Curigliano et al. 2017). An ICC greater than 0.8 was considered to indicate almost perfect agreement.

### Extraction and dimensionality reduction of radiomics features

The VOIs of the tumor, peritumor, and BPE regions were subjected to feature extraction using Pyradiomics version 2.1.2 (Griethuysen et al. 2017). Depending on the registration of the sequences, the T1WI, T1 + C images, and silhouette images can have the same VOIs. Six categories of radiomics features were extracted (first-order, shape, gray-level concurrence matrix [GLCM], gray-level run-length matrix [GLRLM], gray-level size zone matrix [GLSZM], and gray-level co-occurrence matrix [GLDM]) and 1132 features were included in these six categories. There were 3396 radiomics features from the three regions (tumor, peritumor, and BPE) in each sequence, so each patient had 10,188 radiomics features from the three sequences. The Supplementary Materials provide further details of the feature extraction algorithms.

R software version 4.3.1 was used for reduction of feature dimensionality. First, univariate analysis with the univariate rank sum test was used to analyze highly repeatable and significantly correlated features. Then, correlation analysis was performed on the features extracted from the intratumoral, peritumoral, and BPE areas, and highly redundant features (correlation coefficient > 0.6) were deleted. Finally, to prevent overfitting, elastic net logistic regression was used to filter important modeling features with the following specific formula for the cost function:

$$Cost\left( W \right) = \sum\limits_{i = 1}^{N} {\left( {y_{i} - W^{T} x_{i} } \right)^{2} + \lambda \alpha \left\| W \right\|_{1} + \frac{{\lambda \left( {1 - \alpha } \right)}}{2}} \left\| W \right\|_{2}^{2}$$

where Y is the variable to be predicted, W is the weight to be calculated, λ is the penalty term, X is the input feature matrix, and α is the weight of the two error terms (L1 and L2).

### Establishment of an optimal radiomics signature

Three independent radiomics signatures were constructed using multiple logistic regression with tenfold cross-validation and based on the optimal features of the intratumoral, peritumoral, and BPE regions in the training group. Then, the optimal features of these regions were combined in pairs using multiple logistic regression to construct joint radiomics signatures. In addition, the same method was used to fuse the best features of the three regions to construct a mixed joint-signature of all the three regions (Intra-Peri-BPE). The score of each case calculated from these signatures reflects the probability of pCR and was named the “rad-score.” The predictive performance of these radiomics signatures in the training set and the validation set were evaluated using receiver operating characteristic (ROC) curves. Finally, the best signature was selected to construct the prediction model.

### Construction and analysis of machine learning models

**Fig. 2:**
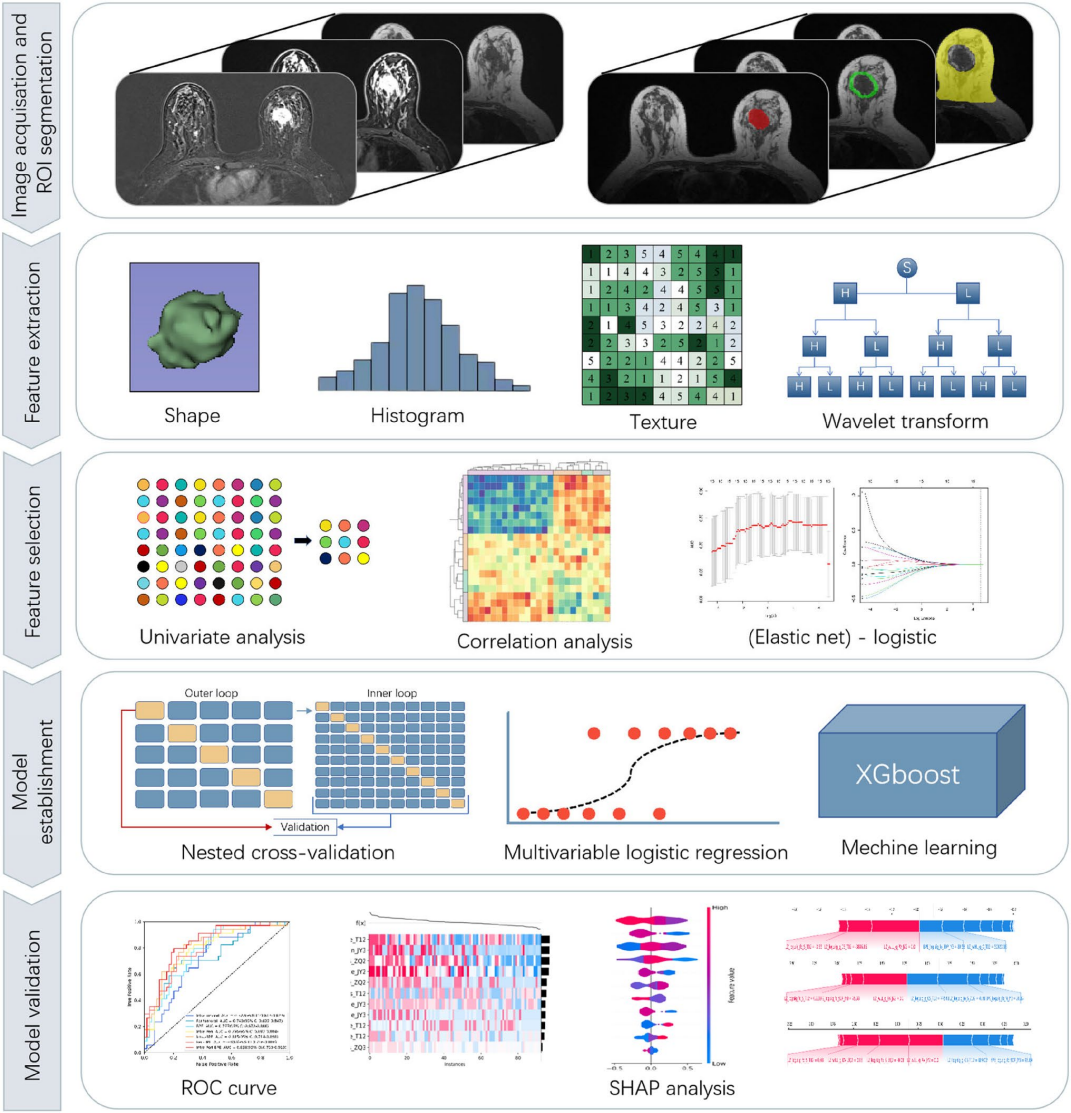
Procedures used for acquisition of MR images, feature extraction, feature selection, model establishment, and model validation

Based on the radiomics features of the optimal signature, the logistic regression (LR), support vector machine (SVM), random forest (RF), K-nearest neighbor (KNN), Bayesian, and extreme gradient boosting (XGBoost) algorithms were used to develop the machine learning models (Fig. 2). Each type of model was based on the training set and used a nested cross-validation procedure that consisted of two nested loops: an outer loop had a repeating stratified random split of the training cohort with 50 repetitions to evaluate classification performance and an inner loop had 5 passes of cross-validation to optimize the hyperparameters of the algorithm. One model was created for each stratified random split, resulting in 50 models. Finally, the model with the highest accuracy in the test group was selected for further use. Then, based on the test group, the diagnostic performance of different machine learning models was verified using ROC curves, and values were compared using the DeLong test. Finally, the machine learning model with the best AUC value was selected. SHapley additive explanation (SHAP) was also used to analyze the relationship between features and outputs in the machine learning models (Rodríguez-Pérez and Bajorath 2020). The Supplementary Materials provide additional details of the procedures used for machine learning and SHAP.

### Statistical analysis

Statistical analyses were performed using SPSS version 24.0, MedCalc version 11.2, R version 4.3.1, and Python version 3.7.3. The Kolmogorov–Smirnov test was used to test the normality of continuous variables. Variables with normal distributions were expressed as means ± standard deviations (SDs) and were compared using the independent samples *t* test; variables with non-normal distributions were expressed as means and quartiles and compared using the Mann–Whitney U test. Categorical variables were expressed as numbers and percentages and compared using the Chi-square test. ROC curves, with calculations of area under the curve (Griethuysen et al. 2017), sensitivity, and specificity, were used to evaluate the predictive performance of the different models. All statistics were two sided, and a *P* value below 0.05 was considered statistically significant.

## Results

### Patient characteristics

We included data on 133 women (mean age: 50.50 years; age range: 25–85 years) who had biopsy-proven primary invasive BCa without distant metastases and who completed a standard NACT regimen (Table 1). Thirty-four of the 93 patients in the training set (36.60%) achieved pCR, and 15 of the 42 patients in the validation set (37.5%) achieved pCR. Comparison of all data in the training set and validation set indicated no significant differences (all *P* > 0.05). In the training set and the validation set, ER, PR, KI67, breast cancer subtypes, and the rad-score were statistically different between the PCR group and the non-PCR group (all *P* < 0.05). In the training set, Her-2 and N stage were also statistically different between the PCR group and the non-PCR group (both *P* < 0.05). However, the rest of the clinical data had no statistical difference (all *P* > 0.05).

**Table 1:** Characteristics of the training set and the validation set

Characteristic			Training set (*n* = 93)				Validation set (*n* = 40)				Training *vs.* Validation
			Total cohort	Non-pCR (*n* = 59)	pCR (*n* = 34)	*P* value	Total cohort	Non-pCR (*n* = 25)	pCR (*n* = 15)	*P* value	*P* value
Outcome		Non-pCR	59 (63.4%)	59 (100.0%)	0 (0.0%)	0.078	25 (62.5%)	25 (100.0%)	0 (0.00%)	0.251	0.806
		pCR	34 (36.6%)	0 (0.0%)	34 (100.0%)		15 (37.5%)	0 (0.0%)	15 (100.0%)		
Age (years)			51.51 ± 10.42	52.20 ± 10.32	49.56 ± 10.08	0.053	48.15 ± 12.08	48.28 ± 13.37	47.93 ± 10.00	0.931	0.107
Menstrual status	Premenopausal		49 (52.7%)	27 (45.8%)	22 (64.7%)	0.703	22 (55.0%)	12 (48.0%)	10 (66.7%)	0.332	0.686
	Postmenopausal		44 (47.3%)	32 (54.2%)	12 (35.3%)		18 (45.0%)	13 (52.0%)	5 (33.3%)		
T stage	T1–2		69 (74.2%)	43 (72.9%)	26 (76.5%)	0.808	31 (77.5%)	19 (76.0%)	12 (80.0%)	> 0.999	0.512
	T3–4		24 (25.8%)	16 (27.1%)	8 (23.5%)		9 (22.5%)	6 (24.0%)	3 (20.0%)		
N stage	N0		26 (28.0%)	17 (28.8%)	9 (26.5%)	0.003*	9 (22.5%)	3 (12.0%)	6 (40.0%)	0.057	0.682
	N1		67 (72.0%)	42 (71.2%)	25 (73.5%)		31 (77.5%)	22 (88.0%)	9 (60.0%)		
BI-RADS stage	3		1 (1.1%)	0 (0.0%)	1 (2.9%)	0.066	1 (2.5%)	0 (0.0%)	1 (6.7%)	0.246	0.339
	4		5 (5.4%)	1 (1.7%)	4 (11.8%)		5 (12.5%)	3 (12.0%)	2 (13.3%)		
	5		54 (58.1%)	35 (59.3%)	19 (55.9%)		23 (57.5%)	17 (68.0%)	6 (40.0%)		
	6		33 (35.5%)	23 (39.0%)	10 (29.4%)		11 (27.5%)	5 (20.0%)	6 (40.0%)		
ER status	Negative		36 (38.7%)	16 (27.1%)	20 (58.8%)	0.002*	17 (42.5%)	6 (24.0%)	11 (73.3%)	0.003*	0.866
	Positive		57 (61.3%)	43 (72.9%)	14 (41.2%)		23 (57.5%)	19 (76.0%)	4 (26.7%)		
PR status	Negative		41 (44.1%)	19 (32.2%)	22 (64.7%)	< 0.001*	17 (42.5%)	7 (28.0%)	10 (66.7%)	0.024*	0.388
	Positive		52 (55.9%)	40 (67.8%)	12 (35.3%)		23 (57.5%)	18 (72.0%)	5 (33.3%)		
HER2 status	Negative		53 (57.0%)	43 (72.9%)	10 (29.4%)	0.001*	26 (65.0%)	19 (76.0%)	7 (46.7%)	0.089	0.151
	Positive		40 (43.0%)	16 (27.1%)	24 (70.6%)		14 (35.0%)	6 (24.0%)	8 (53.3%)		
Ki-67 status	Negative		30 (32.3%)	26 (44.1%)	4 (11.8%)	0.003*	8 (20.0%)	8 (32.0%)	0(0.0%)	0.016*	0.220
	Positive		63 (67.7%)	33 (55.9%)	30 (88.2%)		32 (80.0%)	17 (68.0%)	15 (100.0%)		
Subtype	Luminal A		9 (9.7%)	9 (15.3%)	0(0.0%)	< 0.001*	3 (7.5%)	3 (12.0%)	0(0.0%)	0.006*	0.902
	Luminal B/HER2 −		26 (28.0%)	22 (37.3%)	4 (11.8%)		13 (32.5%)	10 (40.0%)	3(20.0%)		
	Luminal B/HER2 +		22 (23.7%)	11 (18.6%)	11 (32.4%)		9 (22.5%)	7 (28.0%)	2 (13.3%)		
	HER2 + /Non-luminal		19 (20.4%)	6 (10.2%)	13 (38.2%)		6(15.0%)	0 (0.0%)	6 (40.0%)		
	Triple negative		17 (18.3%)	11 (18.6%)	6 (17.6%)		9 (22.5%)	5 (20.0%)	4 (26.7%)		

BI-RAD, Breast Imaging Reporting and Data System; pCR, pathological complete response; ER, estrogen receptor; PR, progesterone receptor; HER2, human epidermal growth factor receptor 2

### Construction and comparison of radiomics signatures in different regions

After reduction of feature dimensionality, there are 4 features in the intratumoral region, 11 features in the peritumoral region, and 8 features in the BPE region. In addition, on combining different regions, there were 15 features from the Intra-Peri regions, 12 features from the Intra-BPE regions, 18 features from the Peri-BPE regions, and 23 features from the Intra-Peri-BPE regions (Supplementary materials).

**Fig. 3:**
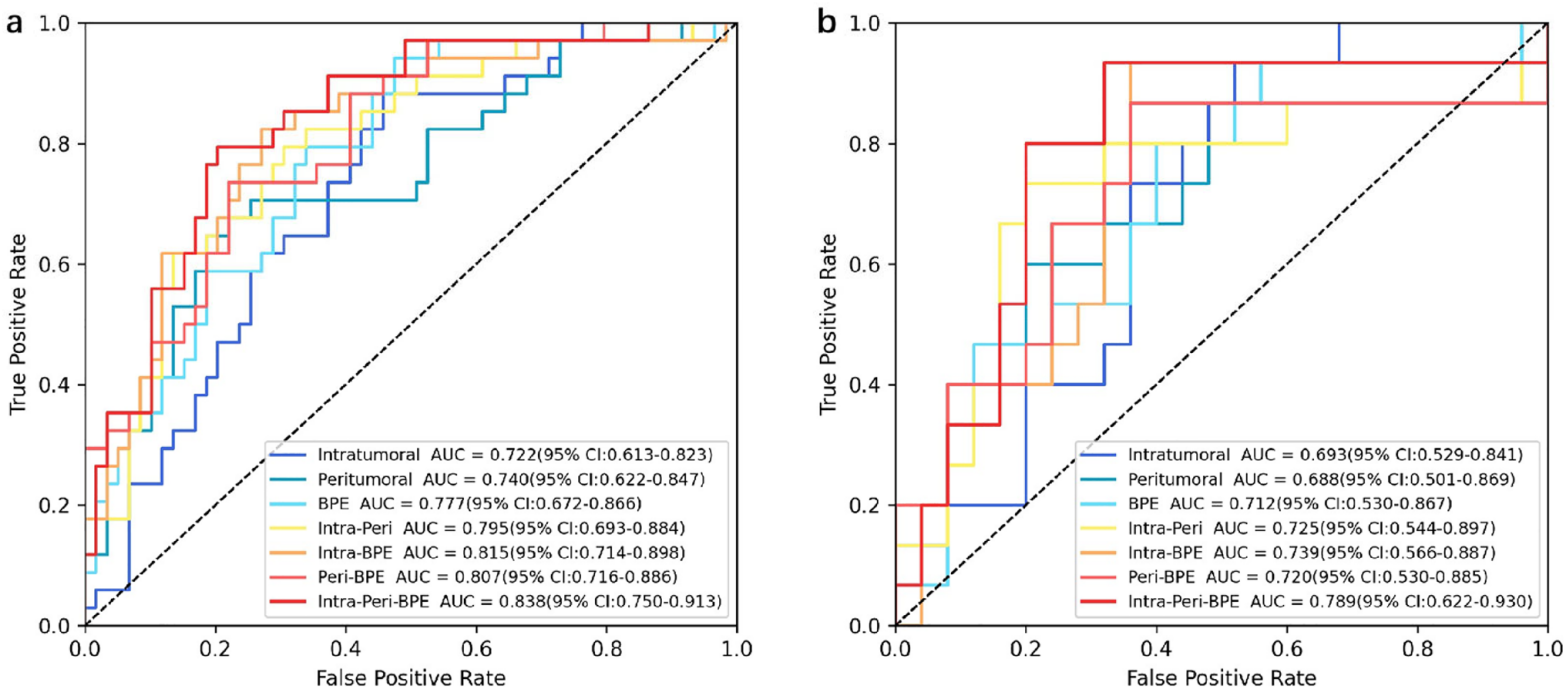
ROC curves for radiomics signatures from different individual regions and combinations of regions in the training set (**a**) and the validation set (**b**)

We constructed radiomics signatures using different individual regions and combinations of regions, and then used ROC analysis to assess the diagnostic performance of all these signatures in the training set and validation set (Fig. 3, Table 2). Among signatures for a single region, BPE had the best predictive value; in addition, adding BPE to the Intra-Peri signature improved the diagnostic performance. The best signature was from all the three regions (Intra-Peri-BPE). The Intra-Peri-BPE signature had an AUC of 0.838 (training set) and 0.789 (test set), an accuracy of 0.796 (training set) and 0.775 (test set), a sensitivity of 0.794 (training set) and 0.733 (test set), and a specificity of 0.797 (training set) and 0.800 (test set).

**Table 2:** Diagnostic performance of radiomics signatures from different individual regions and combinations of regions in the training set and the validation set

Group	Region	AUC (95%CI)	Accuracy	Sensitivity	Specificity
Training	Intratumoral	0.722 (0.613, 0.823)	0.667	0.882	0.542
	Peritumoral	0.740 (0.622, 0.847)	0.753	0.647	0.814
	BPE	0.777 (0.672, 0.866)	0.677	0.941	0.525
	Intra-Peri	0.795 (0.693, 0.884)	0.731	0.794	0.695
	Intra-BPE	0.815 (0.714, 0.898)	0.763	0.824	0.729
	Peri-BPE	0.807 (0.716, 0.886)	0.763	0.735	0.780
	Intra-Peri-BPE	0.838 (0.750, 0.913)	0.796	0.794	0.797
Validation	Intratumoral	0.693 (0.529, 0.841)	0.625	0.733	0.56
	Peritumoral	0.688 (0.501, 0.869)	0.650	0.667	0.640
	BPE	0.712 (0.530, 0.867)	0.600	0.933	0.400
	Intra-Peri	0.725 (0.544, 0.897)	0.625	0.800	0.520
	Intra-BPE	0.739 (0.566, 0.887)	0.675	0.733	0.640
	Peri-BPE	0.720 (0.530, 0.885)	0.675	0.733	0.640
	Intra-Peri-BPE	0.789 (0.622, 0.930)	0.775	0.733	0.800

BPE, background parenchymal enhancement; Intra, intratumoral; Peri, peritumoral

### Construction and validation of machine learning models

**Fig. 4:**
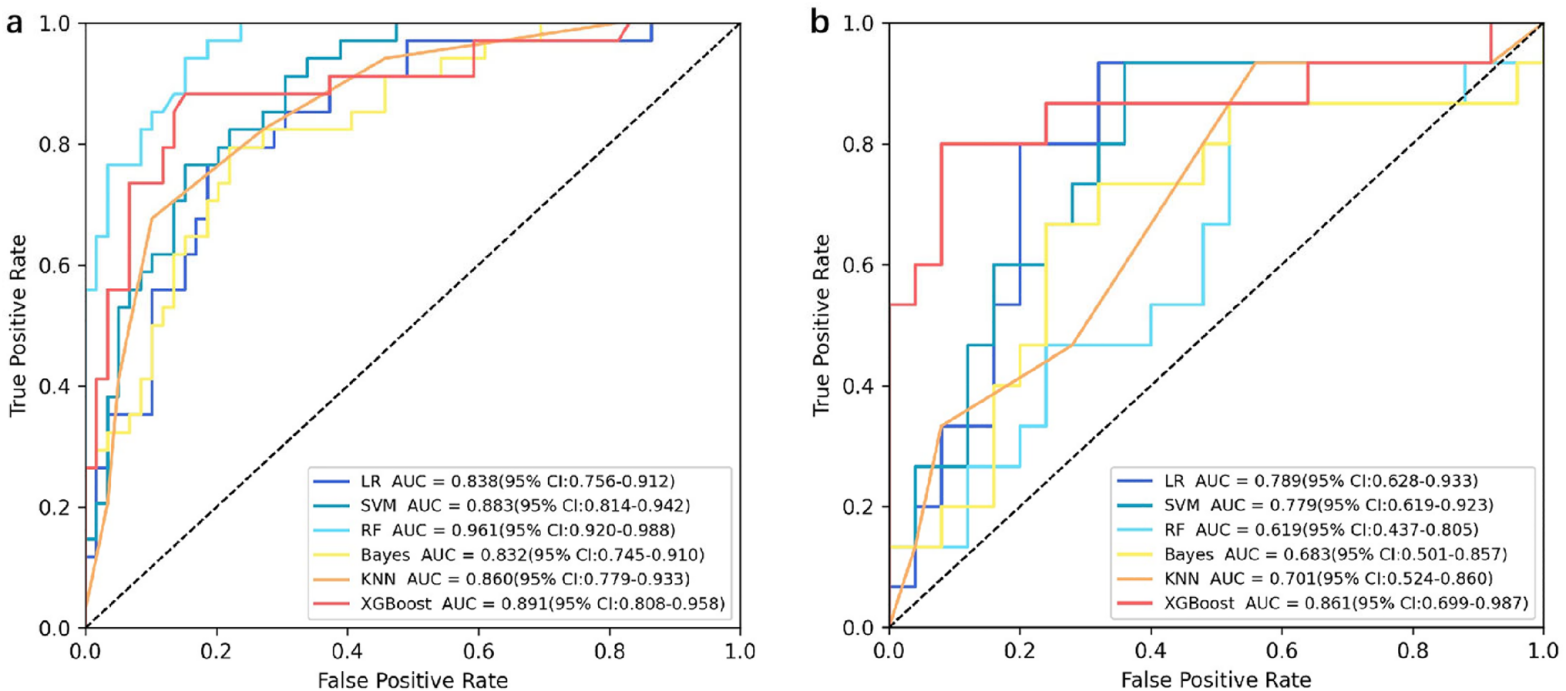
ROC curves for six different machine learning models in the training set (**a**) and the validation set (**b**)

Based on the best radiomics signature model, we used six methods of machine learning to improve model performance (Fig. 4, Table 3). The results show that the XGBoost model had the best performance. Its AUC was 0.891 (training set) and 0.861 (test set), accuracy was 0.86 (training set) and 0.825 (test set), sensitivity was 0.882 (training set) and 0.8 (test set), and specificity was 0.847 (training set) and 0.84 test set).

**Table 3:** Diagnostic performance of six different machine learning models in the training set and the validation set

Group	Model	AUC (95% CI)	Accuracy	Sensitivity	Specificity
Training	LR	0.838 (0.756, 0.912)	0.796	0.794	0.797
	SVM	0.883 (0.814, 0.942)	0.817	0.765	0.847
	RF	0.961 (0.920, 0.988)	0.882	0.941	0.847
	Bayesian	0.832 (0.745, 0.910)	0.785	0.794	0.78
	KNN	0.860 (0.779, 0.933)	0.817	0.676	0.898
	XGBoost	0.891 (0.808, 0.958)	0.86	0.882	0.847
Validation	LR	0.789 (0.628, 0.933)	0.775	0.733	0.8
	SVM	0.779 (0.619, 0.923)	0.725	0.733	0.72
	RF	0.619 (0.437, 0.805)	0.6	0.8	0.48
	Bayesian	0.683 (0.501, 0.857)	0.675	0.733	0.64
	KNN	0.701 (0.524, 0.860)	0.625	0.467	0.72
	XGBoost	0.861 (0.699, 0.987)	0.825	0.8	0.84

LR, logistic regression; SVM, support vector machine; RF, random forest; KNN, K-nearest neighbor; XGBoost, extreme gradient boosting

### Visualization by SHAP

**Fig. 5:**
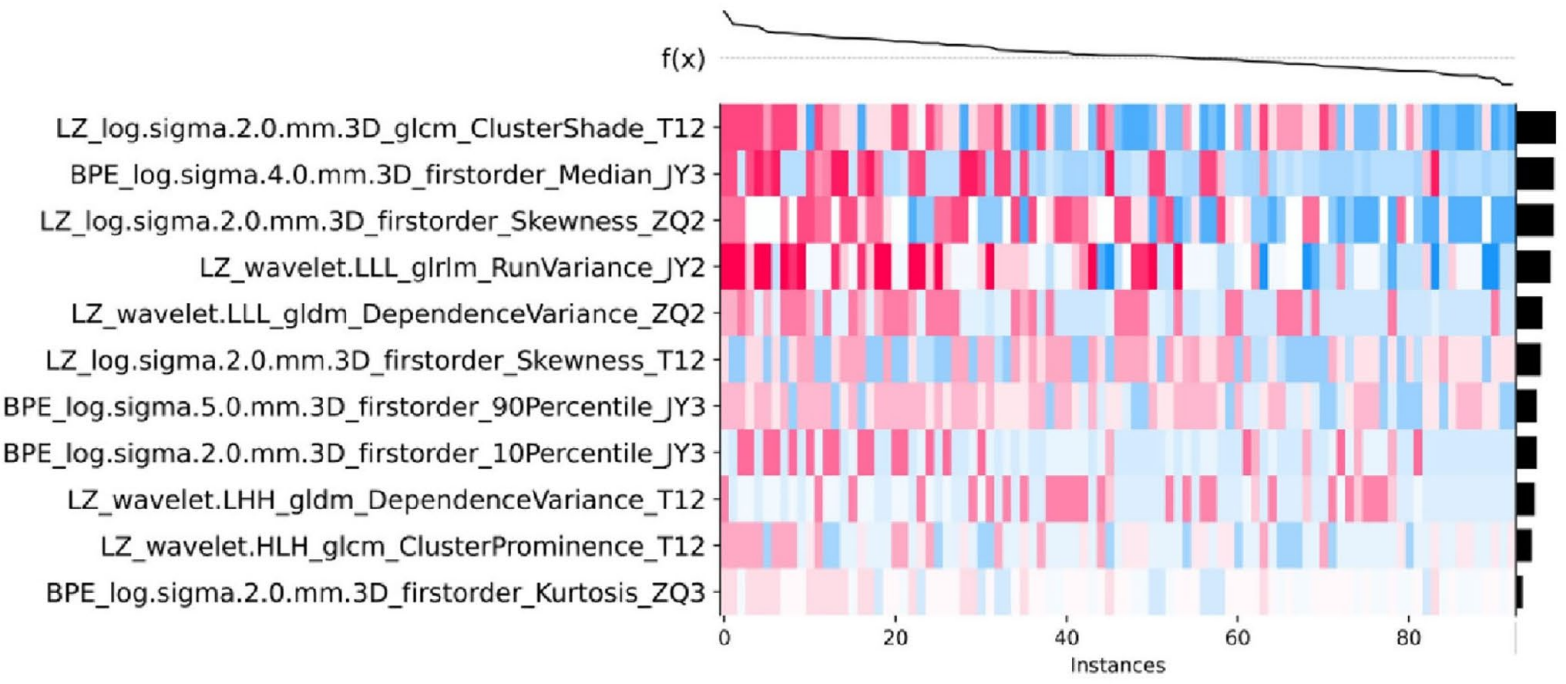
Contribution of each feature (left column) to the prediction of pCR of different patients (horizontal axis) in the GXBoost model. The contribution of each feature is proportional to the amount of red in the heatmap

**Fig. 6:**
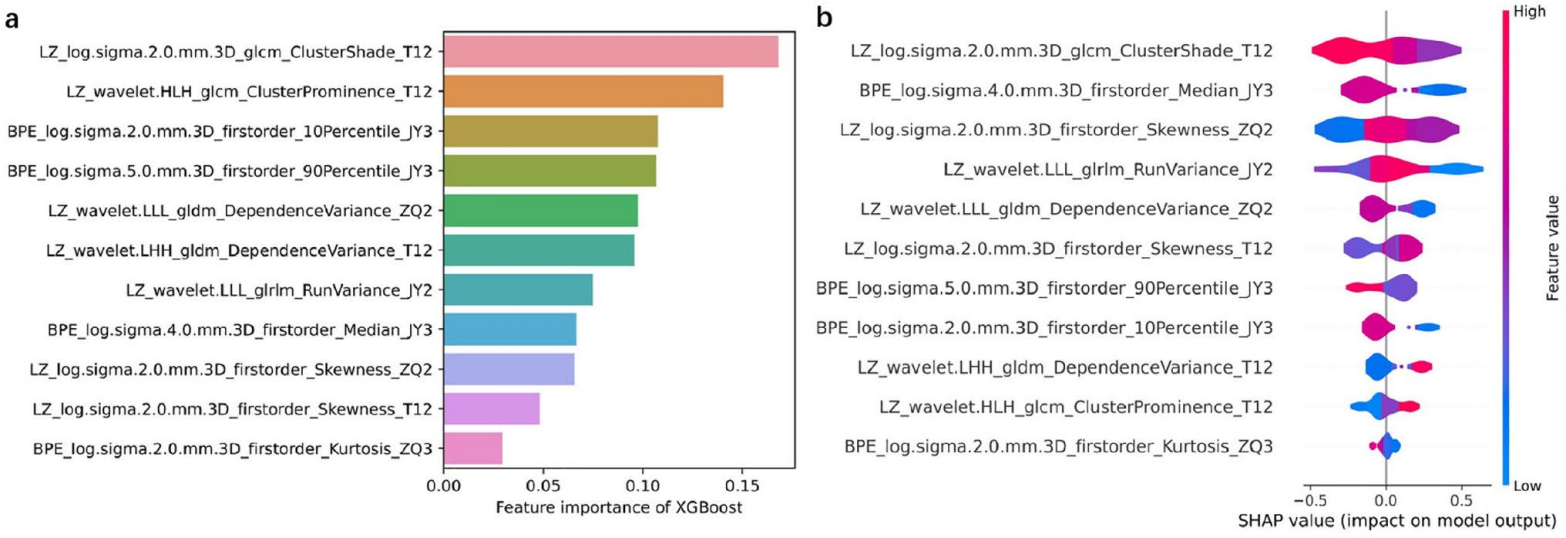
**a** Importance of different radiomics features in the XGBoost model. **b** Distribution of SHAP values (horizontal axis) for radiomics features (vertical axis), with each point representing a sample. A deeper red indicates a larger value of a feature, and a deeper blue indicates a smaller value. Points to the left of the central vertical line have SHAP values less than zero and a negative impact on prediction; points to the right of the central vertical line have SHAP greater than zero and a positive impact on prediction

**Fig. 7:**
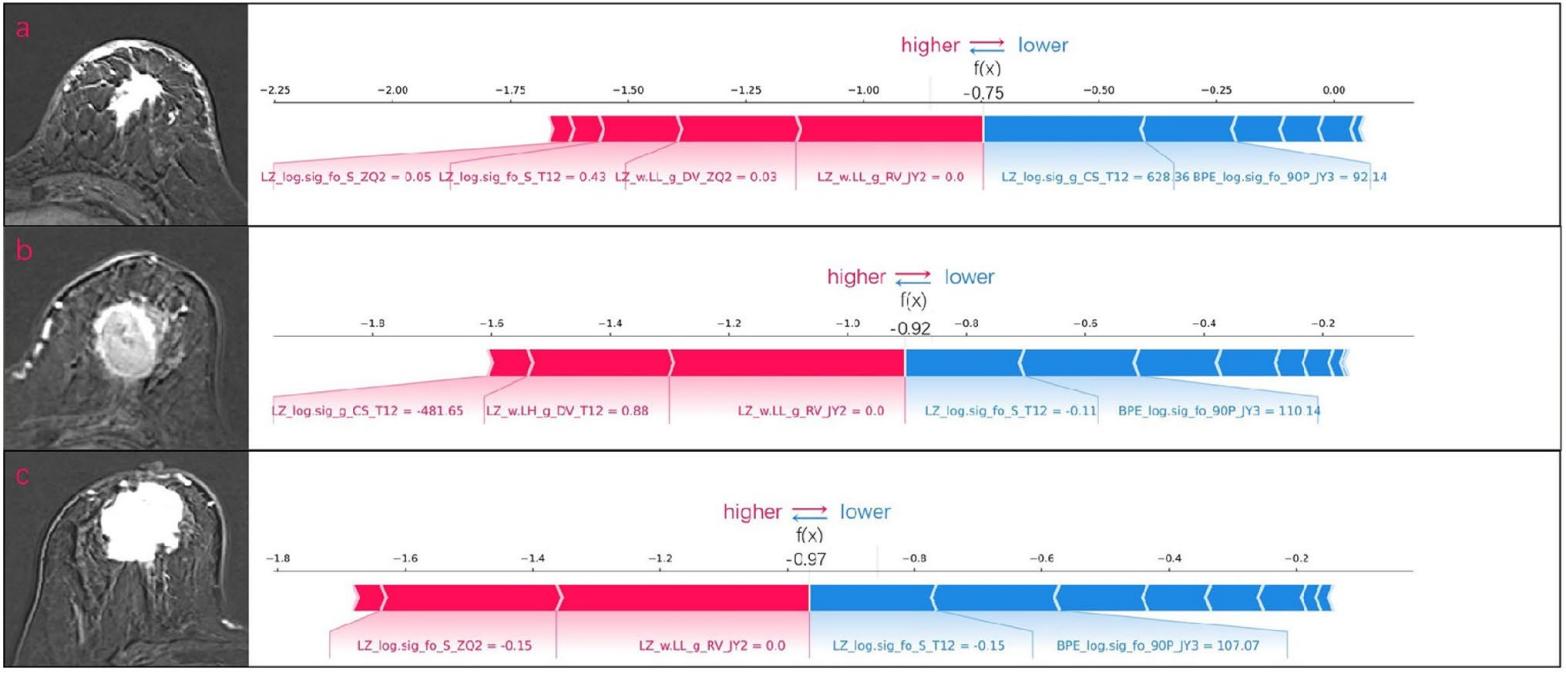
SHAP force plots of three representative patients. The Shapley values of different features are presented as forces that increase or decrease the predicted outcome. Each prediction started with a base value, which is the average Shapley value of all predicted features (− 0.85). The length of each thick colored arrow is proportional to a feature’s contribution to the prediction, with red arrows having a positive effect and blue arrows having a negative effect. **a** A 50-year-old woman with a left breast mass, BI-RADS category 5, biopsy-proven invasive cancer, and HER2 + /non-luminal subtype who received six cycles of docetaxel and trastuzumab treatment. The model predicted pCR, consistent with the pathological result. **b** A 67-year-old woman with a left breast mass, BI-RADS category 5, biopsy-proven invasive cancer, and triple-negative subtype who received four cycles of epirubicin and cyclophosphamide, and then four cycles of paclitaxel. The model predicted non-pCR, consistent with the pathological results. **c** A 52-year-old woman with a right breast mass, BI-RADS category 6, biopsy-proven invasive cancer, and HER2 + /non-luminal subtype who received six cycles of docetaxel and trastuzumab. The model predicted non-pCR, consistent with the pathological results

We then used SHAP for visualization and interpretation of the 11 important features in the XGBoost model and constructed a heatmap to show the contribution of each feature to the prediction (Fig. 5). The results show that LZ_log.sigma.2.0.mm.3D_glcm_ClusterShade_T12 made the strongest contribution to model prediction (Fig. 6). Finally, we constructed SHAP force plots to demonstrate how to interpret these results in three randomly selected patients (Fig. 7). For each patient, these results show the effect of different features on the model’s prediction of clinical outcome.

## Discussion

Our major results are that use of a radiomics signature based on the BPE region provided better predictions of pCR than signatures from the intratumoral region and the peritumoral region, and that addition of the BPE region to the intratumoral and the peritumoral regions improved predictions of the XGBoost model. These results demonstrate that the peritumoral microenvironment, as indicated by BPE, has greater prognostic value than that of the tumor parenchymal area. In addition, a radiomics model based on the combined use of the intratumoral, peritumoral, and BPE regions provided reliable predictions of pCR when using the XGBoost machine learning model. These results confirm the importance of machine learning for prediction of pCR for women with invasive BCa without distant metastases who completed a standard NACT regimen (Radak et al. 2023).

MRI, especially functional imaging such as dynamic contrast-enhanced MRI (DCE-MRI) and diffusion weighted imaging (Curigliano et al. 2017), is playing an increasingly important role in the diagnosis and treatment of BCa. In particular, clinicians use these imaging results to evaluate the morphology, signal strength, and enhancement of the tumor area (Negrão et al. 2019; Lother et al. 2023; Calabrese et al. 2021). However, these classical indicators do not fully reflect the biological behavior of lesions, and they only have limited use for evaluations of treatment response. Therefore, a key direction of recent research has been to obtain more biological information from multi-level and multi-angle omics data to achieve more accurate predictions of tumor status and response to treatment. Radiomics is a new omics method that uses medical imaging results with different algorithms as a non-invasive method to evaluate response to treatment and predict treatment outcome in patients with BCa (Conti et al. 2021). The results of the present study confirmed the usefulness of a radiomics model that combines data from the intratumoral, peritumoral, and BPE regions for prediction of pCR in BCa patients. We also found that the use of all three of these regions provided better predictions than any single region alone. This suggests that the jointly constructed signature provides more comprehensive information and more accurately reflects the biological characteristics and microenvironment of these tumors.

Compared with traditional imaging studies, our radiomics model provided more objective, accurate, and comprehensive information for predicting pCR in BCa patients who received NACT (Liu et al. 2019), and this improvement was due to our use of a combination of radiomics signatures of the peritumoral and BPE regions. Previous studies showed that the radiomics features of the peritumoral region reflected the response of surrounding tissues, such as inflammation and fibrosis. Therefore, the features of the peritumoral region have high specificity in predicting pCR in patients with BCa (Hussain et al. 2021). Our results are therefore consistent, in that the predictive performance of the peritumoral region was greater than that of the intratumoral region. Our novel finding is that the predictive value of the BPE was higher than that of the peritumoral region, presumably due to the unique characteristics of the BPE. The BPE represents the peritumoral microenvironment, and this signal is related to microvessel density and local blood flow in the breast tissue. Thus, BPE may have a high predictive value because a greater enhancement represents an environment that is more favorable for tumor development, growth, and metastasis (Liao et al. 2020). Similar studies have confirmed that BPE is an important predictor of neoadjuvant chemotherapy response. For example, Vignesh A Arasu et al. combined FVT and BPE to predict pCR. The associated BPE area under the curve (Griethuysen et al. 2017) was 0.77 (95% CI 0.56–0.98) (Arasu et al. 2020). This is similar to our result of 0.777 (95% CI 0.672, 0.866). However, predictive performance has no substantial additive improvement when adding BPE to an FTV model. On the contrary, in our study, the diagnostic performance of the intratumoral and peritumoral regions improved after adding the BPE signature. This may be because radiomics features better reflect the heterogeneity of the BPE region. In addition, we also found that radiomics signatures in the tumor region had the lowest predictive value. This may be because the complexity and heterogeneity of tumor tissues lead to unstable predictions. However, we also found that adding the BPE region and the peritumoral region in the joint model significantly improved predictions, and that the BPE region had a greater impact than the peritumoral region. This demonstrates that the peritumoral microenvironment contains more information related to tumor occurrence, development, and prognosis after treatment. Our SHAP analysis also showed that the most important features of the prediction model were from the peritumoral and BPE regions, consistent with our finding that the individual diagnostic value of the peritumoral region and the BPE region was higher than that of the intratumoral region. Our finding that pairwise combinations of radiomics signatures had greater predictive values than those of individual signatures confirmed the importance of using all the three regions for making the best predictions of pCR.

MRI-based radiomics has been widely used for prediction of pCR in patients with BCa (O'Donnell et al. 2022). Compared with previous studies, our approach has certain advantages. Guo et al. used the radiomics features of DCE-MRI combined with clinical features to predict pCR and reported an AUC value of 0.864 in the test set (Guo et al. 2022), similar to our AUC values (training set: 0.891, validation set: 0.861). However, the performance of this previous prediction model from the sole use of imaging omics features was only 0.842. Our results were better, possibly because we tested a variety of machine learning algorithms, and used the optimal algorithm—XGBoost—to construct a prediction model. Similarly, Tahmassasebi et al. (2019) used a variety of machine learning models to predict pCR in patients with BCa and showed that the XGBoost classifier provided the most stable performance and highest accuracy, similar to our results. Umutlu et al. developed a prediction model for pCR in patients with BCa and reported an AUC of 0.92 (Umutlu et al. 2022), higher than that in our study. However, this previous study used multimodal radiomics features from the fusion of images derived from PET and MRI, whereas we only used MRI results. Thus, when considering costs and simplicity, our approach may be more suitable for clinical practice.

### Limitations

There were still some limitations in this study. First, the sample size was relatively small. We therefore plan follow-up studies with larger samples by cooperation with multiple hospitals to verify the stability and applicability of our model. Second, our research used MRI data alone, and the response of BCa patients to NACT depends on imaging features, as well as gene mutations and clinical characteristics. In future, we will consider building a multi-dimensional prediction model that integrates data from radiomics, genomics, clinical characteristics, and elsewhere to achieve a more accurate prediction of pCR. Finally, due to the small number of patients, we were unable to analyze different BCa subtypes, and this may limit the applicability of our predictive model in clinical practice.

## Conclusion

In conclusion, this study used radiomics data from three different regions of breast tissue and six machine learning algorithms to predict pCR in patients with BCa. Our results provide a theoretical basis for the peritumoral microenvironment affecting the efficacy of NACT for these patients. We suggest that future research should explore the biological mechanism by which the peritumoral microenvironment affects the response to NACT, because this may facilitate the identification of new therapeutic targets for BCa and the development of more individualized treatments.

### Supplementary Information

Below is the link to the electronic supplementary material.

**Supplementary file 1:** (DOCX 621 KB)

## Data Availability

The datasets generated during and/or analyzed during the current study are available from the corresponding author on reasonable request.

## Abbreviations

BCa: Breast cancer
NACT: Neoadjuvant chemotherapy
pCR: Pathological complete response
BPE: Background parenchymal enhancement
MRI: Magnetic resonance imaging
T1WI: T1-weighted images
T1 + C: Enhanced T1-weighted images
ROC: Receiver operating characteristic
AUC: Area under the curve
T: Tumor
N: Node
ER: Estrogen receptor
PR: Progesterone receptor
HER-2: Human epidermal growth factor receptor-2
VOI: Volume of interest
ITK: The Insight Segmentation and Registration Toolkit
SPM: Statistical parametric mapping
GLCM: Gray-level concurrence matrix
GLRLM: Gray-level run-length matrix
GLSZM: Gray-level size zone matrix
GLDM: Gray-level co-occurrence matrix
ICC: Intra-class correlation coefficient
PET/CT: Positron emission tomography-computed tomography
DCE-MRI: Dynamic contrast-enhanced magnetic resonance imaging
