# Supplementary material for: Predicting pathological complete response to neoadjuvant chemotherapy in breast cancer patients: use of MRI radiomics data from three regions with multiple machine learning algorithms
Source: J Cancer Res Clin Oncol. 2024 Mar 21;150(3):147. doi: 10.1007/s00432-024-05680-y (PMC10957588; doi:10.1007/s00432-024-05680-y)
Supplement: Supplementary file 1 — (DOCX 621 KB) [file 432_2024_5680_MOESM1_ESM.docx]

**Supplementary Materials**

1. **Clinical and pathological information**

**1.1 NAC Regimen and Criteria for pCR**

According to the National ComprehensiveS Cancer Network Guidelines(1), all patients received the standard six to eight cycles of NACT before surgery. HER-2 negative patients received 4 cycles of epirubicin and cyclophosphamide treatment, followed by 4 cycles of paclitaxel treatment. HER-2 positive patients received 6 cycles of docetaxel and trastuzumab treatment, and all patients underwent surgical treatment after NACT. The surgical specimens were fixed in 10% neutral buffered formalin and overnight processing in a standard tissue processor. Slides are cut at 5μm and dyed in an automatic dyeing system. Histopathological examinations and analyses are performed by pathologists with at least 12 years of experience in breast pathology who turn a blind eye to MRI data. Specimens were pathologically assessed using the Miller-Payne system(2). pCR was defined as the absence of residual invasive tumors (Miller-Payne grade 5, possible presence of residual ductal carcinoma in situ), the absence of lymph node infiltration in the ipsilateral sentinel lymph nodes or lymph nodes removed during axillary dissection (ypT0/isN0).

**1.2 Definition of** **Miller-Payne grade system**

Gread 1: No change or some alteration to individual malignant cells but no reduction in overall cellularit.

Gread 2: A minor loss of tumour cells but overall cellularity still high, and up to 30% loss.

Gread 3: Between an estimated 30% and 90% reduction in tumour cell.

Gread 4: A marked disappearance of tumour cells such that only small clusters or widely dispersed individual cells remain, and more than 90% loss of tumour cells.

Gread 5: No malignant cells identiﬁable in sections from the site of the tumour, only vascular fibroelastotic stroma remains often containing macrophages. However, ductal carcinoma in situ(DCIS) may be present.

**1.3 Immunohistochemical Evaluation**

Immunohistochemistry (IHC) was used to evaluate the expression of several receptors and antigens commonly associated with BCa from biopsy in the pre NACT, including estrogen receptor (ER), progesterone receptor (PR), antigen Ki67 (Ki-67)antigen Ki67 (Ki-67) and human epidermal growth factor receptor 2 (HER2). Tumors with nuclear staining<1% indicate ER/PR negative, while tumors with staining ≥ 1% indicate ER/PR positive. Next, 20% was established as the cutoff value for Ki-67 expression. Regarding HER2 expression, IHC scores of 0 or 1+ were denoted as HER2-negative, and 3+ was denoted as HER2-positive. An IHC score of 2+ required further investigation using in situ hybridization (ISH), with non-amplified results denoted as HER2-negative and amplified as HER2-positive.

**1.4 Breast cancer subtype**

The molecular subtype was categorized according to the 2017 St. Gallen guidelines. Patients were classified into 5 groups of cancer subtypes according to the 2017 St. Gallen guidelines(3): Luminal A, Luminal B HER2 negative, Luminal B HER2 positive, HER2 positive non luminal, triple negative.

**2.** **MRI scan**

All patients were scanned using a 3.0T MRI scanner (Skyra; Siemens Healthineers) with a 16-channel body coil, and all patients were scanned in the prone position. The routine sequences included T2-weighted imaging (T2WI), T1-weighted imaging (T1WI), diffusion-weighted imaging, and dynamic contrast-enhanced (DCE). We used post-processing workstation to acquire subtraction images. The detailed parameters for each sequence were illustrated in **Table S1**. Before injecting contrast agent, obtain initial fat saturated T1WI plain scan, and then use a high-pressure syringe to inject Gd-DTPA intravenously at a flow rate of 0.2mmol/Kg and 2ml/s. Collect the first post contrast scan 2 minutes after injecting contrast agent. Collect four subsequent contrast-enhanced images at 90 second intervals, with each patient having five stages of contrast-enhanced images (t=2, 3.5, 5, 6.5, and 8 minutes).

**Table S1.** MRI parameters of each sequence

| **Scanner** | **Sequence** | **Orientation** | **TR**  **(ms)** | **TE**  **(ms)** | **FOV**  **(mm^2^)** | **Thickness**  **(mm)** | **Interslice gap**  **(mm)** | **Matrix** |
| --- | --- | --- | --- | --- | --- | --- | --- | --- |
| SIEMENS 3.0T  (Skyra) | T_2_WI | Sagittal | 6060 | 90 | 180×180 | 3 | 0.6 | 320×224 |
|  | T_2_WI | Axial | 4790 | 134 | 200×200 | 3 | 0.6 | 384×451 |
|  | T_1_WI | Axial | 662 | 9.6 | 180×180 | 3 | 0.6 | 320×224 |
|  | DWI | Axial | 7330 | 56.0 | 200×200 | 3 | 0.8 | 112×100 |
|  | T_1_CE | Axial | 616 | 9.6 | 180×180 | 3 | 0.6 | 320×224 |

Note. TR, repetition time; TE, echo time; FOV, field of view.

**3. Image preprocessing and segmentation**

Image segmentation program is as follows. Firstly, the SPM toolkit in Matlab software was used to rigorously register the images of T1WI, enhanced third-phase T1-weighted (T1+C) sequences and dynamic contrast-enhanced subtraction images in order to reduce the potential influence of the parameters of a scanning scheme. After that, the standardized T1WI images were imported into the ITK software to manually segment the entire tumor layer by layer and to determine the volume of interest (VOI). Since the three sequences have been rigorously registered, tumor VOI obtained from T1WI can be applied directly to other sequences. All cases undertook the same VOI segmentation method.

Image preprocessing including resample, intensity normalization and gray-level discretization were performed with the Pyradiomics program. To be more specific, image preprocessing was performed by resampling the images with a resolution of 1×1×1 mm3 through the linear interpolation method and by discretizing and normalizing the image gray level to order 32. Extracted texture features were standardized, which removed the unit limits of the data of each feature and converted it into a dimensionless pure value. This allowed the indexes of different units or orders to be compared and weighted. We used a z-score normalization to make the image intensities fit a standard normal distribution with $\mu\text{=0}$ and $\sigma\text{=1}$, where $\mu$ is the mean value of the images, and $\sigma$ is the standard deviation. The normalized values (also called z-scores) of the image intensities (x) were calculated as follows:

$$z=\frac{x-\mu}{\sigma}$$

**4. Details of Dimension reduction**

We targeted three regions for dimensionality reduction, including intratumoral, peritumoral and BPE. Each region contained a total of 3396 features from three sequences. First, each region was screened for significant features, and we used the univariate rank sum test, with P<0.05 as a significant difference, to find significantly related features. Then, we performed non-redundant feature screening on the remaining features, and we used correlation analysis between features to delete highly redundant radiomics features with correlation greater than 0.6 for the remaining features of intratumoral, peritumoral and BPE regions, respectively. Finally, an elastic net logistic model was used to filter important modeling features for the remaining features. The specific formula is as follows:

$$Cost\left(W\right)=\sum_{i=1}^{N} \left(y_{i}-W^{T}x_{i}\right)^{2}+\lambda\alpha\left\|W\right\|_{1}+\frac{\lambda\left(1-\alpha\right)}{2}\left\|W\right\|_{2}^{2}$$

After screening, there were 4 remaining features in the tumor region, 11 remaining features in the peritumoral region, and 8 remaining features in the BPE region. In addition, we performed different combinations of these regions, and again used the Elastic net-logistic model to screen the features of these combined regions. Finally, 15 features were left in the tumor combined with peritumoral region, 12 features were left in the tumor combined with BPE, 18 features were left in the peritumoral combined with BPE, and 23 features were left in the combination of the three regions. The specific features and elastic network calculation process are shown in **Figure S1**, **Figure S2** and **Table S2**.


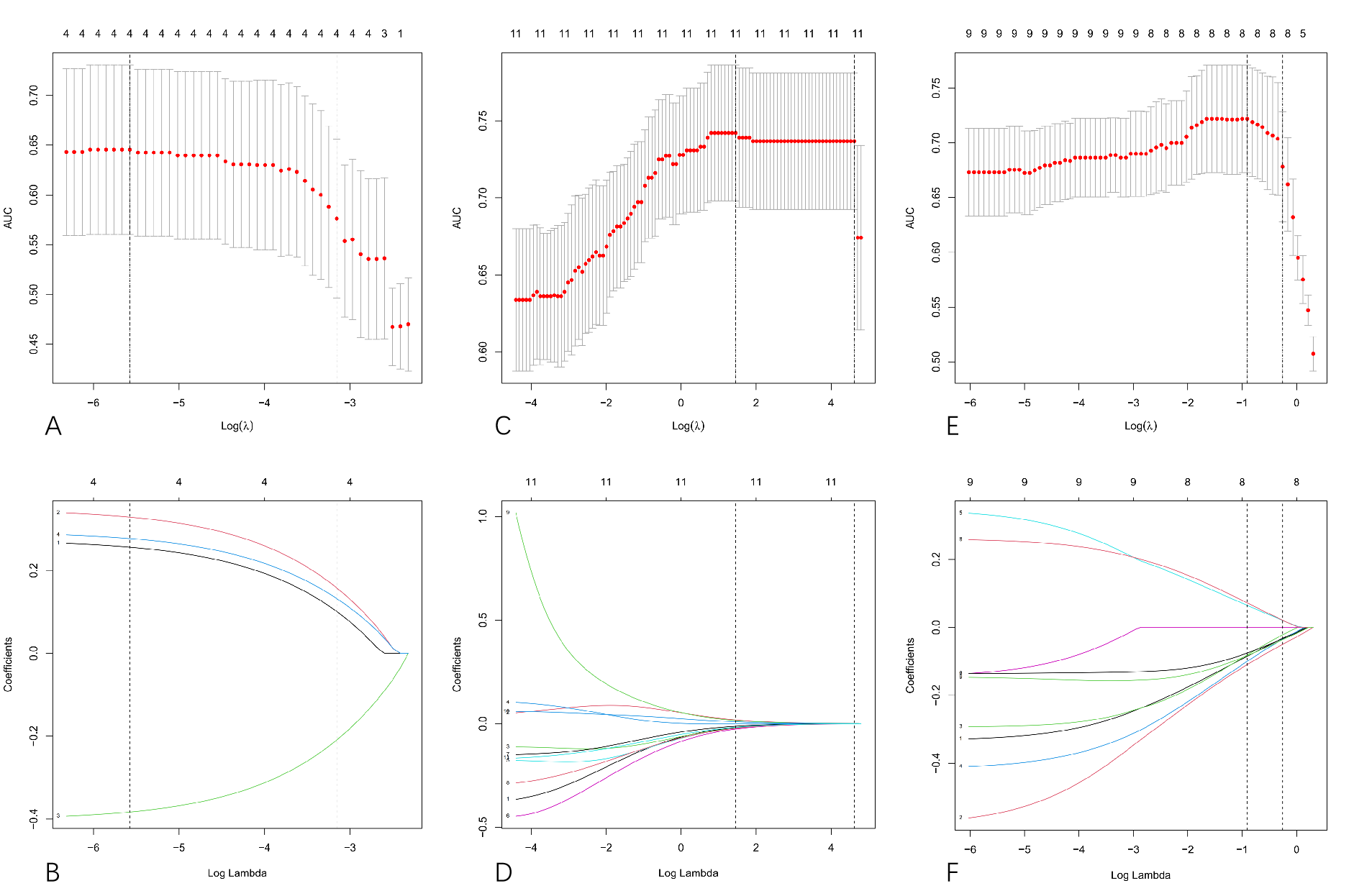


**Figure S1**. Dimension reduction process using (Elastic net) - logistic. A and B The dimension reduction process of intratumoral, log(λ)=-5.574, corr=0.6, α=1; C and D The dimension reduction process of peritumoral, log(λ)=1.454, corr=0.6, α=0; E and F The dimension reduction process of BPE, log(λ)=-0.906, corr=0.6,α=0.1.


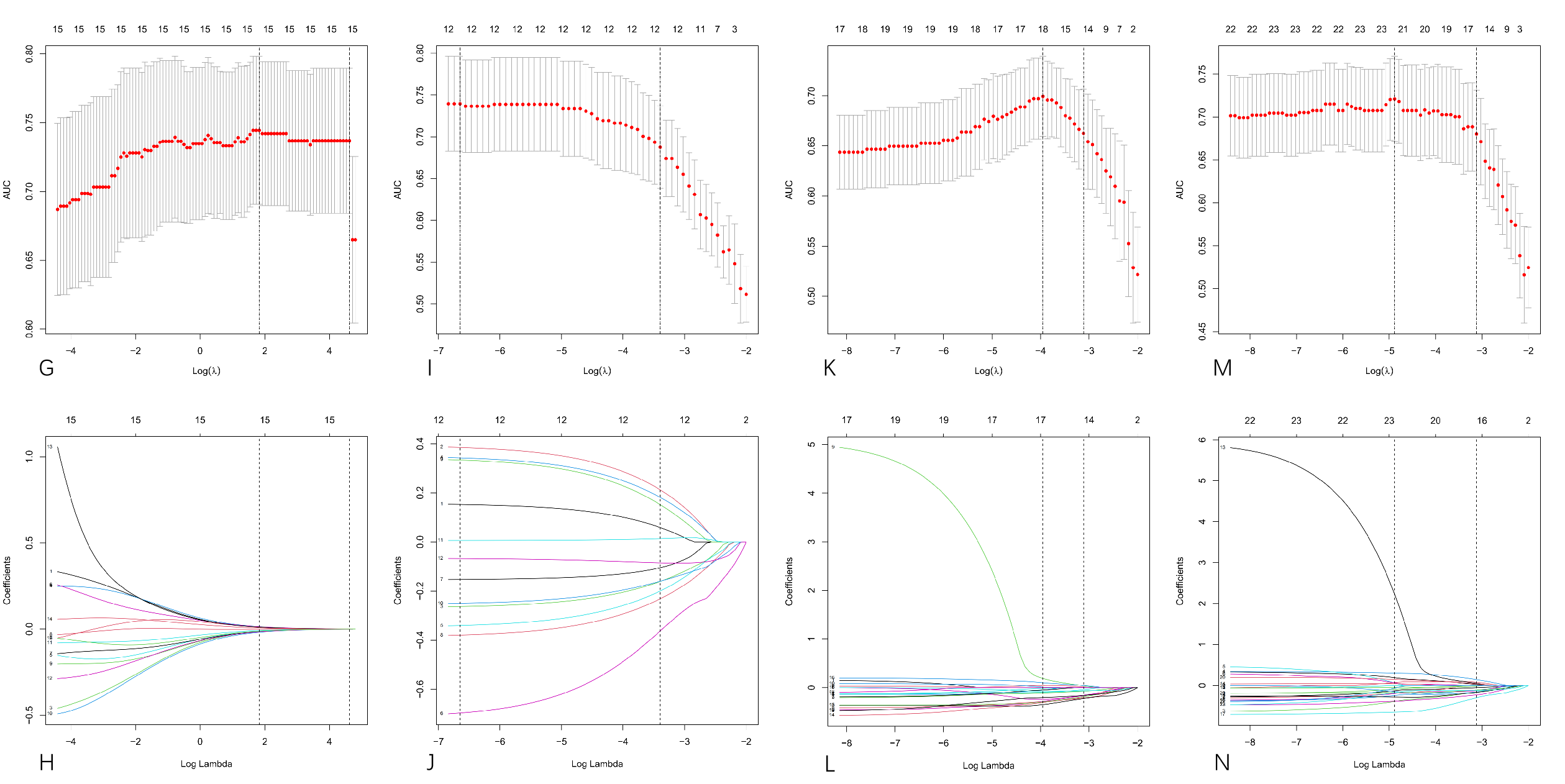


**Figure S2**. Dimension reduction process using (Elastic net) - logistic. G and H The dimension reduction process of intra-peri, log(λ)=1.826, corr=0.6, α=0; I and J The dimension reduction process of intra-BPE, log(λ)=-6.651, corr=0.6, α=1; K and L The dimension reduction process of peri-BPE, log(λ)=-3.953, corr=0.6, α=1; M and N The dimension reduction process of intra-peri-BPE, log(λ)=-3.953, corr=0.6, α=1.

**Table S2** details of remaining features by (Elastic net) - logistic regression analysis

|  | feature | Coefficient | OR |
| --- | --- | --- | --- |
| intratumoral | wavelet.LHH_glcm_Correlation_T11 | -0.38308631 | 0.681754055 |
|  | log.sigma.3.0.mm.3D_gldm_LargeDependenceLowGrayLevelEmphasis_JY1 | 0.256195265 | 1.292004986 |
|  | wavelet.HHH_firstorder_Median_ZQ1 | 0.276974482 | 1.319132708 |
|  | wavelet.LLL_firstorder_Minimum_JY1 | 0.328778404 | 1.389269965 |
| peritumoral | log.sigma.2.0.mm.3D_glcm_ClusterShade_T12 | -0.025897897 | 0.974434577 |
|  | wavelet.LHH_gldm_DependenceVariance_T12 | -0.022087775 | 0.978154374 |
|  | log.sigma.2.0.mm.3D_firstorder_Skewness_T12 | -0.021884371 | 0.978353354 |
|  | wavelet.LLL_firstorder_Kurtosis_JY2 | -0.021191659 | 0.979031307 |
|  | log.sigma.3.0.mm.3D_firstorder_Median_JY2 | -0.018914126 | 0.981263624 |
|  | wavelet.LLL_gldm_DependenceVariance_ZQ2 | -0.017477989 | 0.982673865 |
|  | log.sigma.4.0.mm.3D_glcm_Idmn_T12 | -0.012520249 | 0.987557803 |
|  | wavelet.LLL_glrlm_RunVariance_JY2 | -0.001577152 | 0.998424091 |
|  | log.sigma.2.0.mm.3D_firstorder_Skewness_ZQ2 | 0.008757734 | 1.008796195 |
|  | wavelet.HLH_glcm_ClusterProminence_T12 | 0.015695579 | 1.015819401 |
|  | wavelet.LLL_firstorder_10Percentile_JY2 | 0.018382563 | 1.018552563 |
| BPE | log.sigma.4.0.mm.3D_firstorder_Median_JY3 | -0.109595033 | 0.896196992 |
|  | wavelet.LHH_glszm_HighGrayLevelZoneEmphasis_JY3 | -0.099930203 | 0.904900575 |
|  | log.sigma.2.0.mm.3D_firstorder_10Percentile_JY3 | -0.085004467 | 0.918508181 |
|  | wavelet.LHH_firstorder_Kurtosis_ZQ3 | -0.083211357 | 0.920156645 |
|  | log.sigma.5.0.mm.3D_firstorder_90Percentile_JY3 | -0.081162656 | 0.922043703 |
|  | log.sigma.2.0.mm.3D_firstorder_Kurtosis_ZQ3 | -0.075621476 | 0.927167095 |
|  | wavelet.LHL_firstorder_RootMeanSquared_T13 | 0.064594322 | 1.066726189 |
|  | wavelet.LLH_glszm_SmallAreaEmphasis_ZQ3 | 0.071874128 | 1.074520084 |
| Intra-peri | LZ_log.sigma.2.0.mm.3D_glcm_ClusterShade_T12 | -0.018439822 | 0.981729151 |
|  | LZ_wavelet.LHH_gldm_DependenceVariance_T12 | -0.015717075 | 0.984405794 |
|  | LZ_log.sigma.2.0.mm.3D_firstorder_Skewness_T12 | -0.01557619 | 0.984544492 |
|  | tumor_wavelet.LHH_glcm_Correlation_T11 | -0.015336852 | 0.984780158 |
|  | LZ_wavelet.LLL_firstorder_Kurtosis_JY2 | -0.015057918 | 0.985054885 |
|  | LZ_log.sigma.3.0.mm.3D_firstorder_Median_JY2 | -0.013121817 | 0.986963899 |
|  | LZ_wavelet.LLL_gldm_DependenceVariance_ZQ2 | -0.012382714 | 0.987693636 |
|  | LZ_log.sigma.4.0.mm.3D_glcm_Idmn_T12 | -0.008637805 | 0.991399393 |
|  | LZ_wavelet.LLL_glrlm_RunVariance_JY2 | -0.001321871 | 0.998679002 |
|  | LZ_log.sigma.2.0.mm.3D_firstorder_Skewness_ZQ2 | 0.006392665 | 1.006413141 |
|  | LZ_wavelet.HLH_glcm_ClusterProminence_T12 | 0.011132192 | 1.011194385 |
|  | tumor_log.sigma.3.0.mm.3D_gldm_LargeDependenceLowGrayLevelEmphasis_JY1 | 0.011463358 | 1.011529314 |
|  | tumor_wavelet.LLL_firstorder_Minimum_JY1 | 0.011980423 | 1.012052475 |
|  | LZ_wavelet.LLL_firstorder_10Percentile_JY2 | 0.013076752 | 1.013162627 |
|  | tumor_wavelet.HHH_firstorder_Median_ZQ1 | 0.013410443 | 1.013500766 |
| Intra-BPE | BPE_log.sigma.4.0.mm.3D_firstorder_Median_JY3 | -0.696781343 | 0.498186217 |
|  | BPE_wavelet.LHH_glszm_HighGrayLevelZoneEmphasis_JY3 | -0.379146896 | 0.684445063 |
|  | BPE_log.sigma.2.0.mm.3D_firstorder_10Percentile_JY3 | -0.340385017 | 0.711496332 |
|  | tumor_wavelet.LHH_glcm_Correlation_T11 | -0.263225639 | 0.768568459 |
|  | BPE_log.sigma.2.0.mm.3D_firstorder_Kurtosis_ZQ3 | -0.250022739 | 0.778783074 |
|  | BPE_log.sigma.5.0.mm.3D_firstorder_90Percentile_JY3 | -0.152766154 | 0.858330416 |
|  | BPE_wavelet.LHH_firstorder_Kurtosis_ZQ3 | -0.067789702 | 0.934456967 |
|  | BPE_wavelet.LLH_glszm_SmallAreaEmphasis_ZQ3 | 0.005827214 | 1.005844226 |
|  | tumor_log.sigma.3.0.mm.3D_gldm_LargeDependenceLowGrayLevelEmphasis_JY1 | 0.152432897 | 1.164664307 |
|  | BPE_wavelet.LHL_firstorder_RootMeanSquared_T13 | 0.33361556 | 1.39600636 |
|  | tumor_wavelet.HHH_firstorder_Median_ZQ1 | 0.342360707 | 1.408268177 |
|  | tumor_wavelet.LLL_firstorder_Minimum_JY1 | 0.384190626 | 1.468425335 |
| Peri-BPE | BPE_log.sigma.4.0.mm.3D_firstorder_Median_JY3 | -0.345949995 | 0.707547867 |
|  | BPE_log.sigma.5.0.mm.3D_firstorder_90Percentile_JY3 | -0.301752782 | 0.739520865 |
|  | BPE_wavelet.LHH_glszm_HighGrayLevelZoneEmphasis_JY3 | -0.290507231 | 0.747884122 |
|  | LZ_wavelet.LHH_gldm_DependenceVariance_T12 | -0.282316333 | 0.75403512 |
|  | BPE_log.sigma.2.0.mm.3D_firstorder_10Percentile_JY3 | -0.279900423 | 0.755859004 |
|  | LZ_log.sigma.2.0.mm.3D_glcm_ClusterShade_T12 | -0.233681521 | 0.791613888 |
|  | BPE_wavelet.LHH_firstorder_Kurtosis_ZQ3 | -0.201430426 | 0.817560457 |
|  | LZ_wavelet.LLL_firstorder_Kurtosis_JY2 | -0.108364391 | 0.897300569 |
|  | LZ_wavelet.LLL_gldm_DependenceVariance_ZQ2 | -0.095436102 | 0.908976441 |
|  | BPE_log.sigma.2.0.mm.3D_firstorder_Kurtosis_ZQ3 | -0.075911045 | 0.926898655 |
|  | LZ_wavelet.LLL_glrlm_RunVariance_JY2 | -0.0495787 | 0.951630262 |
|  | LZ_log.sigma.4.0.mm.3D_glcm_Idmn_T12 | -0.048919919 | 0.952257384 |
|  | LZ_log.sigma.2.0.mm.3D_firstorder_Skewness_T12 | -0.016130418 | 0.98399898 |
|  | BPE_wavelet.LLH_glszm_SmallAreaEmphasis_ZQ3 | 0.001821034 | 1.001822693 |
|  | LZ_log.sigma.2.0.mm.3D_firstorder_Skewness_ZQ2 | 0.009605299 | 1.009651578 |
|  | LZ_wavelet.LLL_firstorder_10Percentile_JY2 | 0.044575104 | 1.045583501 |
|  | BPE_wavelet.LHL_firstorder_RootMeanSquared_T13 | 0.105049895 | 1.110766031 |
|  | LZ_wavelet.HLH_glcm_ClusterProminence_T12 | 0.192282728 | 1.212013138 |
| Intra-peri-BPE | wavelet.LHH_glcm_Correlation_T11 | -0.38308631 | 0.681754055 |
|  | log.sigma.3.0.mm.3D_gldm_LargeDependenceLowGrayLevelEmphasis_JY1 | 0.256195265 | 1.292004986 |
|  | wavelet.HHH_firstorder_Median_ZQ1 | 0.276974482 | 1.319132708 |
|  | wavelet.LLL_firstorder_Minimum_JY1 | 0.328778404 | 1.389269965 |
|  | log.sigma.2.0.mm.3D_glcm_ClusterShade_T12 | -0.025897897 | 0.974434577 |
|  | wavelet.LHH_gldm_DependenceVariance_T12 | -0.022087775 | 0.978154374 |
|  | log.sigma.2.0.mm.3D_firstorder_Skewness_T12 | -0.021884371 | 0.978353354 |
|  | wavelet.LLL_firstorder_Kurtosis_JY2 | -0.021191659 | 0.979031307 |
|  | log.sigma.3.0.mm.3D_firstorder_Median_JY2 | -0.018914126 | 0.981263624 |
|  | wavelet.LLL_gldm_DependenceVariance_ZQ2 | -0.017477989 | 0.982673865 |
|  | log.sigma.4.0.mm.3D_glcm_Idmn_T12 | -0.012520249 | 0.987557803 |
|  | wavelet.LLL_glrlm_RunVariance_JY2 | -0.001577152 | 0.998424091 |
|  | log.sigma.2.0.mm.3D_firstorder_Skewness_ZQ2 | 0.008757734 | 1.008796195 |
|  | wavelet.HLH_glcm_ClusterProminence_T12 | 0.015695579 | 1.015819401 |
|  | wavelet.LLL_firstorder_10Percentile_JY2 | 0.018382563 | 1.018552563 |
|  | log.sigma.4.0.mm.3D_firstorder_Median_JY3 | -0.109595033 | 0.896196992 |
|  | wavelet.LHH_glszm_HighGrayLevelZoneEmphasis_JY3 | -0.099930203 | 0.904900575 |
|  | log.sigma.2.0.mm.3D_firstorder_10Percentile_JY3 | -0.085004467 | 0.918508181 |
|  | wavelet.LHH_firstorder_Kurtosis_ZQ3 | -0.083211357 | 0.920156645 |
|  | log.sigma.5.0.mm.3D_firstorder_90Percentile_JY3 | -0.081162656 | 0.922043703 |
|  | log.sigma.2.0.mm.3D_firstorder_Kurtosis_ZQ3 | -0.075621476 | 0.927167095 |
|  | wavelet.LHL_firstorder_RootMeanSquared_T13 | 0.064594322 | 1.066726189 |
|  | wavelet.LLH_glszm_SmallAreaEmphasis_ZQ3 | 0.071874128 | 1.074520084 |

**Table S3.**The information of radiomics features

| Feature Groups (N) | Feature names | Feature Groups (N) | Feature names |
| --- | --- | --- | --- |
|  | firstorder_10Percentile |  | glszm_GrayLevelNonUniformity |
|  | firstorder_90Percentile |  | glszm_GrayLevelNonUniformityNormalized |
|  | firstorder_Energy |  | glszm_GrayLevelVariance |
|  | firstorder_Entropy |  | glszm_HighGrayLevelZoneEmphasis |
|  | firstorder_InterquartileRange |  | glszm_LargeAreaEmphasis |
|  | firstorder_Kurtosis |  | glszm_LargeAreaHighGrayLevelEmphasis |
|  | firstorder_Maximum |  | glszm_LargeAreaLowGrayLevelEmphasis |
| First-order features (N = 18) | firstorder_MeanAbsoluteDeviation | GLSZM texture features | glszm_LowGrayLevelZoneEmphasis |
|  | firstorder_Mean | (N = 16) | glszm_SizeZoneNonUniformity |
|  | firstorder_Median |  | glszm_SizeZoneNonUniformityNormalized |
|  | firstorder_Minimum |  | glszm_SmallAreaEmphasis |
|  | firstorder_Range |  | glszm_SmallAreaHighGrayLevelEmphasis |
|  | firstorder_RobustMeanAbsoluteDeviation |  | glszm_SmallAreaLowGrayLevelEmphasis |
|  | firstorder_RootMeanSquared |  | glszm_ZoneEntropy |
|  | firstorder_Skewness |  | glszm_ZonePercentage |
|  | firstorder_TotalEnergy |  | glszm_ZoneVariance |
|  | firstorder_Uniformity |  |  |
|  | glrlm_GrayLevelNonUniformity |  | glcm_Autocorrelation |
|  | glrlm_GrayLevelNonUniformityNormalized |  | glcm_ClusterProminence |
|  | glrlm_GrayLevelVariance |  | glcm_ClusterShade |
|  | glrlm_HighGrayLevelRunEmphasis |  | glcm_ClusterTendency |
|  | glrlm_LongRunEmphasis |  | glcm_Contrast |
| GLRLM texture features | glrlm_LongRunHighGrayLevelEmphasis | GLCM texture features | glcm_Correlation |
| (N = 16) | glrlm_LongRunLowGrayLevelEmphasis | (N = 24) | glcm_DifferenceAverage |
|  | glrlm_LowGrayLevelRunEmphasis |  | glcm_DifferenceEntropy |
|  | glrlm_RunEntropy |  | glcm_DifferenceVariance |
|  | glrlm_RunLengthNonUniformity |  | glcm_Id |
|  | glrlm_RunLengthNonUniformityNormalized |  | glcm_Idm |
|  | glrlm_RunPercentage_T2 |  | glcm_Idmn |
|  | glrlm_RunVariance |  | glcm_Idn |
|  | glrlm_ShortRunEmphasis |  | glcm_Imc1 |
|  | glrlm_ShortRunHighGrayLevelEmphasis |  | glcm_Imc2 |
|  | glrlm_ShortRunLowGrayLevelEmphasis |  | glcm_InverseVariance |
|  |  |  | glcm_JointAverage |
|  |  |  | glcm_JointEnergy |
|  |  |  | glcm_JointEntropy |
|  |  |  | glcm_MCC |
|  |  |  | glcm_MaximumProbability |
|  |  |  | glcm_SumAverage |
|  |  |  | glcm_SumEntropy |
|  |  |  | glcm_SumSquares |
|  | gldm_DependenceEntropy |  | ngtdm_Busyness |
|  | gldm_DependenceNonUniformity |  | ngtdm_Coarseness |
| GLDM texture features | gldm_DependenceNonUniformityNormalized | NGTDM texture features | ngtdm_Complexity |
| (N = 14) | gldm_DependenceVariance | (N = 5) | ngtdm_Contrast |
|  | gldm_GrayLevelNonUniformity |  | ngtdm_Strength |
|  | gldm_GrayLevelVariance |  |  |
|  | gldm_HighGrayLevelEmphasis |  |  |
|  | gldm_LargeDependenceEmphasis |  |  |
|  | gldm_LargeDependenceHighGrayLevelEmphasis |  |  |
|  | gldm_LargeDependenceLowGrayLevelEmphasis |  |  |
|  | gldm_LowGrayLevelEmphasis |  |  |
|  | gldm_SmallDependenceEmphasis |  |  |
|  | gldm_SmallDependenceHighGrayLevelEmphasis |  |  |
|  | gldm_SmallDependenceLowGrayLevelEmphasis |  |  |
| Los features (N = 186) | Log-sigma-1.0，2.0_* (N =186) |  |  |

Note: GLCM, Gray-level co-occurrence matrices; GLRLM, Gray-level run length matrix; GLSZM, Gray-level size zone matrix; GLDM, Gray-level dependence matrix. *The abbreviated representation of feature types

**Table S4.** The detailed information of remaining radiomics features

| **Sequence** | **Feature** | **Category** |
| --- | --- | --- |
| T_1_WI  (n=11) | wavelet.LHH_glcm_Correlation_T11 | GLCM |
|  | log.sigma.2.0.mm.3D_glcm_ClusterShade_T12 | GLCM |
|  | wavelet.LHH_gldm_DependenceVariance_T12 | GLDM |
|  | log.sigma.2.0.mm.3D_firstorder_Skewness_T12 | First-order |
|  | log.sigma.4.0.mm.3D_glcm_Idmn_T12 | GLCM |
|  | wavelet.HLH_glcm_ClusterProminence_T12 | GLCM |
|  | wavelet.LHL_firstorder_RootMeanSquared_T13 | First-order |
| T_1_WI+C  (n=11) | wavelet.HHH_firstorder_Median_ZQ1 | First-order |
|  | wavelet.LLL_gldm_DependenceVariance_ZQ2 | GLDM |
|  | wavelet.LHH_firstorder_Kurtosis_ZQ3 | First-order |
|  | log.sigma.2.0.mm.3D_firstorder_Kurtosis_ZQ3 | First-order |
|  | wavelet.LLH_glszm_SmallAreaEmphasis_ZQ3 | GLSZM |
| T1jy  (n=10) | log.sigma.3.0.mm.3D_gldm_LargeDependenceLowGrayLevelEmphasis_JY1 | GLDM |
|  | wavelet.LLL_firstorder_Minimum_JY1 | First-order |
|  | wavelet.LLL_firstorder_Kurtosis_JY2 | First-order |
|  | log.sigma.3.0.mm.3D_firstorder_Median_JY2 | First-order |
|  | wavelet.LLL_glrlm_RunVariance_JY2 | GLRLM |
|  | wavelet.LLL_firstorder_10Percentile_JY2 | First-order |
|  | log.sigma.4.0.mm.3D_firstorder_Median_JY3 | First-order |
|  | wavelet.LHH_glszm_HighGrayLevelZoneEmphasis_JY3 | GLSZM |
|  | log.sigma.2.0.mm.3D_firstorder_10Percentile_JY3 | First-order |
|  | log.sigma.5.0.mm.3D_firstorder_90Percentile_JY3 | First-order |

Note. Rad-score of the fusion model can be calculated by intercept and their respective coefficients. intercept = -1.515.

**5. Machine Learning Details**

In our study, we used 6 machine-learning classifiers (Logistic regression, Bayes, XGBoost, K-Nearest neighbors, Random forest and Support vector machines). Nested cross-validation process was used for each type of machine learning modeling (**Figure S3**). At last, we focused on and validated the XGBoost model because of its better performance.


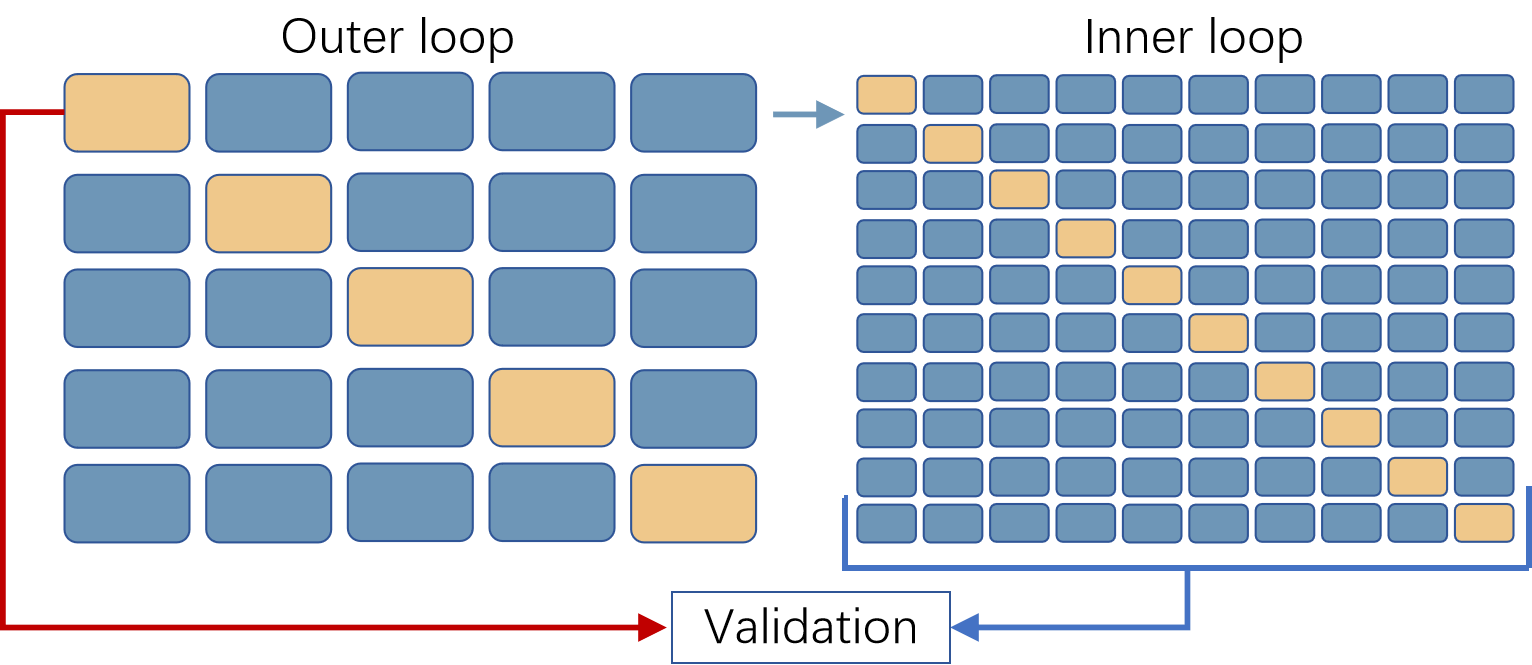


**Figure S3**. Nested cross-validation procedure of machine learning on the training cohort.

**eXtreme Gradient Boosting (XGBoost)**

When dealing with small and medium structured data, the decision tree algorithm is the best. Additionally, XGBoost is an integrated machine-learning algorithm based on decision trees, framed by Gradient Boost. To train the model, we should define the objective function to measure the fitting degree of the model to the training data. The salient characteristic of objective functions is that it consists of training loss and a regularization term. Moreover, the XGBoost technique optimizes both parts of the model. As a result, the XGBoost model provides a more accurate prediction model and effectively prevents overfitting.

**Bayesian (BY)**

Bayesian has the lowest probability of classification errors or the lowest average risk with a predetermined cost among various classifiers. Its design method is the most basic statistical classification method. The classification principle calculates an object's posterior probability using the Bayesian formula based on its prior probability, which is the probability that the object belongs to a particular class. The class with the highest posterior probability is selected as the class to which the object belongs.

**Random forest (RF)**

Random forest is an ensemble machine learning method for classification and regression, which operated by constructing a large number of decision trees and outputting classes as a single tree (classification) or average prediction (regression) model. Random forest provides an improvement to bagging with a modification step of random sampling of predictors.

**Support vector machine (SVM)**

Support vector machine is a machine learning approach which is based on the structural risk minimization principle of statistics learning. It projected data into a multidimensional space and classified it with hyper-planes.

**K-Nearest neighbor (KNN)**

The k-Nearest Neighbor (KNN) classification algorithm is a theoretically mature method and one of the simplest machine learning algorithms. The idea of this method is that in the feature space, if most of the k nearest (i.e. the closest) samples near a sample belong to a particular category, then the sample also belongs to that category.

**Logistic regression** **(LR)**

Logistic regression analysis is a statistical method which is used to analyze a data set in which one or more independent variables that determine the outcome. The outcome was measured by a dichotomous variable, in which there were only two possible outcomes.

**6.** **Visualization of the prediction SHAP (SHapley additive explanation)**

With the increasing arithmetic power of machine learning, models become increasingly complex. An XGBoost model often contains tens or hundreds of boosting trees, which highly reduces the interpretability of the model. To understand the inherent causality of model complexity, SHapley Additive exPlanations (SHAP) was used to analyze the relationship between the features and output in the XGBoost ‘black box’. SHAP analysis evaluated the SHAP value of each feature of the training sample, which represented the sensitivity of that feature to changes in model output. By linearly decomposing the prediction result into the effect of each feature, the importance of features could be calculated and the role of different features in the model could be visualized. And explains the model’s prediction as the sum of the Shapley values of each input feature:

$g\left( x’ \right)=\varphi_{0}+\sum_{M}^{j=1} \varphi_{j}$

where g (x’) is the value of the model, Φ0 is the constant that explains the model (i.e., the predicted mean of all training samples), and Φj is the imputed value (Shapley value) of each feature.

**References**

1. Goetz MP, Gradishar WJ, Anderson BO, et al. NCCN Guidelines Insights: Breast Cancer, Version 3.2018. J Natl Compr Canc Netw 2019;17(2):118-126.

2. Ogston KN, Miller ID, Payne S, et al. A new histological grading system to assess response of breast cancers to primary chemotherapy: prognostic significance and survival. Breast 2003;12(5):320-327.

3. Curigliano G, Burstein HJ, Winer EP, et al. De-escalating and escalating treatments for early-stage breast cancer: the St. Gallen International Expert Consensus Conference on the Primary Therapy of Early Breast Cancer 2017. Ann Oncol 2017;28(8):1700-1712.
